# Autism Spectrum Disorders: A Recent Update on Targeting Inflammatory Pathways with Natural Anti-Inflammatory Agents

**DOI:** 10.3390/biomedicines11010115

**Published:** 2023-01-03

**Authors:** Ramu Singh, Anglina Kisku, Haripriya Kungumaraj, Vini Nagaraj, Ajay Pal, Suneel Kumar, Kunjbihari Sulakhiya

**Affiliations:** 1 Neuro Pharmacology Research Laboratory, Department of Pharmacy, Indira Gandhi National Tribal University, Amarkantak 484887, Madhya Pradesh, India; 2 Department of Kinesiology and Health, School of Art and Sciences, Rutgers, The State University of New Jersey, Piscataway, NJ 08854, USA; 3 Department of Biomedical Engineering, Rutgers, The State University of New Jersey, Piscataway, NJ 08854, USA; 4 Keck Center for Collaborative Neuroscience, Department of Cell Biology and Neuroscience, Rutgers, The State University of New Jersey, Piscataway, NJ 08554, USA; 5 Shriners Hospitals Pediatric Research Center (Center for Neural Rehabilitation and Repair), Lewis Katz School of Medicine, Temple University, Philadelphia, PA 19140, USA

**Keywords:** autism spectrum disorder, inflammatory pathway, neuropsychiatric disorders, natural anti-inflammatory agents

## Abstract

Autism spectrum disorder (ASD) is a heterogeneous category of developmental psychiatric disorders which is characterized by inadequate social interaction, less communication, and repetitive phenotype behavior. ASD is comorbid with various types of disorders. The reported prevalence is 1% in the United Kingdom, 1.5% in the United States, and ~0.2% in India at present. The natural anti-inflammatory agents on brain development are linked to interaction with many types of inflammatory pathways affected by genetic, epigenetic, and environmental variables. Inflammatory targeting pathways have already been linked to ASD. However, these routes are diluted, and new strategies are being developed in natural anti-inflammatory medicines to treat ASD. This review summarizes the numerous preclinical and clinical studies having potential protective effects and natural anti-inflammatory agents on the developing brain during pregnancy. Inflammation during pregnancy activates the maternal infection that likely leads to the development of neuropsychiatric disorders in the offspring. The inflammatory pathways have been an effective target for the subject of translational research studies on ASD.

## 1. Introduction

Autism spectrum disorder (ASD) is a neurodevelopment disorder [1] characterized by an inadequate shortfall in social involvement and social communication across various contexts and restricted areas [2] and stereotype behavior, especially in early childhood [3]. Additionally, male patients are more numerous than females in a 3:1 ratio [4]. The current prevalence is investigated at 1% in the United Kingdom, 1.5% in the United States, and ~0.2% in India [5,6]. According to Freitas et al. (2018), the western nation’s average age of 8 years old has increased by almost 150% between 2000 and 2014, posing a public health concern in North America [7]. Studies from North America, Asia, and Europe indicate a 1–2% moderate prevalence of ASD. Numerous causes, including hereditary and environmental toxins, stress, weakened immunological function, mitochondrial dysfunction, and neuroinflammation, are mentioned in the unidentified pathogenesis [8]. Classic innate failure of metabolism is a monogenic disease that can affect a subgroup of ASD patients, including 12.73% of children suffering from ASD [9,10]. Despite the rise in ASD cases [10], current treatments only partially alleviate some of the symptoms of ASD rather than curing them. Only 2 drugs, risperidone, and aripiprazole have been approved by the Food and Drug Administration of the United States of America for the treatment of disturbing symptoms in ASD patients. However, factors like (1) the condition of the subject, which may affect various aspects of daily function, (2) the significant direct and indirect reasonable effects of treatments, and (3) the suffering experienced by the subject’s entire family highlight the need for ongoing research into effective interventions. Although there have been many classifications and developments about ASD, its genesis is still unknown, and there are few questions about the straightforward nature of the problem [11]. Immunological flaws have been linked to ASD for many years, but they have just reached their pinnacle. The earliest indication of a relationship between the immune system and ASD came from a 1976 study that discovered 5 of 13 autistic infants had undetectable antibody titers despite prior rubella immunization, while every control subject had detectable titers. In the past few decades, research on animal models and human models has revealed evidence of changes in the central and peripheral immune systems’ functioning in ASD, excluding the activation of immune cells, production of autoantibodies, inequality in cytokines and chemokines, and increased permeability of brain regions [12]. Several immunological dysfunction and inflammation in ASD have been proposed as potential targets. Tumor necrosis factor alpha (TNF-α), Interleukin-(IL) 6, and monocytes chemotactic proteins 1 (MCP-1), the most potent of which is also mast cells chemotactic, are recognized as biomarkers of inflammation in the brain and cerebrospinal fluids (CSF) of various autistic individuals [1]. The brain infection makes pro-inflammatory cytokines and chemokines disposable, and they have been linked to hippocampal and cerebral damage [1]. A rich source of a different biomarker is mast cells. Immune cells are stored in mast cells, which can frequently discharge stimulation [13]. The observation of inflammation as a probable etiology factor in psychopathology serves more as an inflammatory condition of brain physiology activity which is an integral part of biological mechanisms and environmental factors [14].

Different search engines such as Science Direct, PubMed, Google Scholar, and Google Patent were used (up to October 2022). Additionally, we looked for methodological work in neuropsychiatric research published during 2005–2022 and collected in online books and article alerts. We incorporated published studies that looked at the prevalence and risk factors for selective reporting, inclusion, or both in systematic reviews of ASD interventions that target various inflammatory pathways. We included empirical studies evaluating any kind of selective reporting or inclusion, such as analyses of the number of pathways targeted, inclusion of biomarkers in systematic reviews, and research based on the findings. Independently, two review authors chose empirical studies for inclusion, extracted the data, and evaluated the risk of bias. Any disagreements regarding the inclusion or exclusion of empirical studies, data extraction, and bias risk were settled by a third review author. We utilized different keywords such as ASD, depression, inflammatory pathway, neuroinflammation, and maternal immune activation for the inclusion and exclusion of studies.

This review summarizes the impact of inflammation during pregnancy that activates the maternal infection and the development of neuropsychiatric disorders such as ASD in the offspring. This review also explores biomarkers and pathology-related mechanisms that most likely regulate the various inflammatory pathways that result in damage to the developing brain. We also examine the effect of increased production of inflammatory cytokines, chemokines, and immune cells in circulation. Next, review of research on genetic variations in cytokine-related brain damage susceptibility is discussed. Finally, we discuss the effective ways to reduce these inflammatory pathways using potential neuroprotective intervention strategies.

## 2. Pathophysiology of ASD

**Figure 1:**
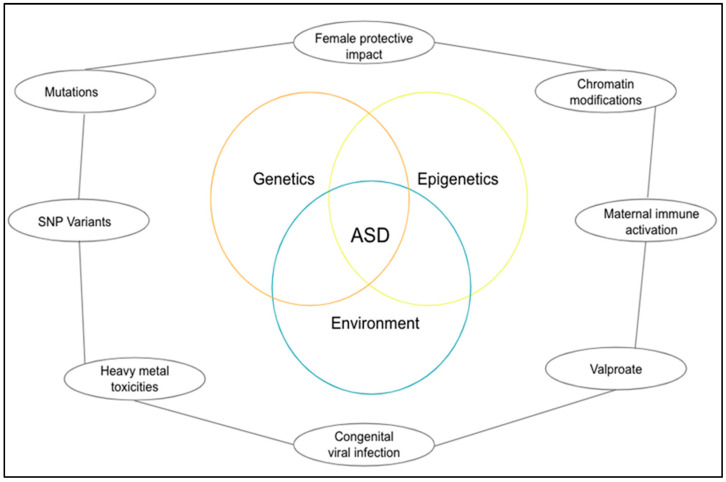
A diagrammatical representation of the pathogenesis of ASD.

Surprisingly research and neuropathological findings in autism have failed to identify anomalies in brain structure. Despite a limited number of postmortem studies, autism does not yet exhibit consistent microscopic neuropathology. The synaptic route is suggested as an etiology factor in ASD by the genetic studies reviewed here (Figure 1), but it is still not entirely clear what the core pathogenesis process is or how it contributes to the emergence of the social and communication deficits distinct from ASD. However, the findings from both in vitro and mutant mouse studies offer a glimpse of some of the molecular processes that may contribute to the emergence of ASD, such as impaired synapse development, connectivity, and stabilization [15].

### 2.1. Genetics

Numerous studies have indicated that there is a genetic component to autism, and genome-wide similar hypotheses have revealed some candidate genes for obsessive behavior. The chance of broad developmental abnormalities resurfacing in children with autism is approximately 2–8%, and it rises to 12–20%. Interestingly, it has been demonstrated that the difference between autistics and the general population is highly heritable, with an uniform genetic influence on autism [16]. Even though the outcomes are diverse. These findings have sparked an effort in studies to attempt and identify the association between genetic and viral infection related to ASD [17,18].

Based on the protection of the female action in ASD, it is currently reported that approximately 10,000 dizygotic two-of-a-kind estimates for autistic features have been verified naturally. Siblings of the female probands who scored population-based 90% or higher above the autistic trait distribution were much more likely to do so than those of the male probands. The findings about copy number of variations in a large number of autistic patients, also suggested that females may be under a more significant burden of familial etiological impact to manifest autistic features than males [19]. A study on Turner syndrome in individuals with only one X chromosome, in which confirmation for an additional X-linked locus for social behavior was acquired, provides another possible illustration of the protective effect, particularly for women, with ASD [20].

There are inheritable methods for examining the inborn cause of ASD, including whole-exome sequencing, genome-wide collaboration investigations, copy-number inequality research, and affinity studies. Most of the modern documentation has paid attention to parental, perinatal, and obstetric factors. Even if it is a rare community, some consequence environmental vulnerabilities provide crucial confirmation for external factors that might significantly increase the chance of ASD. These include rubella infection or the use of drugs like thalidomide or valproate during pregnancy, which have been linked to a 100-fold increase in the chance of autism. In more recent epidemiological research, the risk of ASD has been linked to maternal illness, which can be identified by fever or the flu during pregnancy [21].

### 2.2. Epigenetic

Without changing the DNA sequence, the respective epigenetic process modifies the chromatin structure and regulates the alternate of different genes. Numerous studies have demonstrated the role of poor epigenetic regulation in the genesis of ASD [22]. Patients with ASD showed changes in the genes that code for the proteins used in the epigenetic technique. The revealed mutation results in altered protein expression and behavior, which worsens the ASD behavior problem. After reviewing a database of genes linked to ASD, the scientists concluded that 42 of the 215 genes that cause ASD are directly connected to epigenetic changes in gene expression [20].

A rising number of ASD candidates have genes that have been identified by generative screening as having a major function in epigenetic pathways, which accounts for a sizeable portion of the epigenetic abnormalities in ASD. An increasing body of evidence from studies on humans and animals suggests that ASD and other neurodevelopmental disorders may be influenced by the prenatal environment, in utero environment, and prenatal and early postnatal being [23]. Valproate, a significant environmental risk factor for ASD, inhibits histone deacetylase and interferes with folic acid metabolism. Inadequate folate reduces methyl contribution and ultimately results in a dysfunctional epigenetic regulation in the context of the methylene tetrahydrofolate reductase gene polymorphism [2].

Furthermore, it has been suggested that epigenetic processes can trigger the immune system during pregnancy and can potentially increase sensitivity to autism spectrum disorder. According to this enlarged community theory’s findings, prolonged fever-induced gestation was linked to a threefold increased universality factor for ASD, and maternal influenza microorganisms were associated with a fold increased prevalence of having a kid with autism. Additionally, the findings of the mouse model study of maternal allergic asthma (MAA) suggest that changes in maternal immune activation (MIA) during gestation may raise the risk of autistic disorder in offspring. The offspring of mice with MAA exhibited replacement social and repetitive behavior. As a result of maternal allergic asthma, the researchers set out to modify the methylation region in fetal microglia to increase gene complexity in immune signaling pathways and synaptic circuits. These studies suggest that maternal immune response may affect epigenetic patterns and contribute to the growth of ASD. Many ideas now under consideration examine the role of mRNA in the genesis of ASD and show the variable expression of mRNA in ASD patients [20]. According to research, microRNAs (miRNAs) may be one of the factors contributing to ASD [24]. Small non-coding RNAs called miRNAs control gene expression and are frequently associated with biological functions, including neurodevelopment. miRNA-181c and miRNA-30d are upregulated in VPA (valproic acid) rat model. Additionally, an analysis of the genes containing miRNA-30 and miRNA-181c binding sites was carried out. Numerous genes that are primarily involved in the growth of the nervous system are targeted by miR-NA-30d [24].

### 2.3. Environmental Factors

Environmental factors, however, can clearly affect the development of autism. It is tempting to speculate that the majority of known potential factors, including prenatal stress, prenatal infection, maternal Zn^2+^ deficiency, and the mother’s susceptibility to harmful as well as strongly distinctive factors, will very probably all have different sides to the same story [25].

Literature shows that there are more than 200 chemicals that are powerful teratogens that have neurotoxic effects on an adult brain. A prolonged release of neurotoxin and a significant period of sensitivity of the central nervous system (CNS) negatively affect neurodevelopmental. Exploratory findings suggest that because of the placental barrier’s concentration gradient, extent, and thickness, which are most likely involved in behavioral teratogenesis, particularly with regard to ASD, various chemicals, including heavy metals, air impurities, organophosphates, and halogenated hydrocarbons, diffuse across the placental barrier [26].

#### 2.3.1. Heavy Metal

Lead (Pb) is now well-recognized as being environmentally harmful. Heavy metals have been found in the cord blood, indicating fetal fragility. In addition, mercury (Hg) accumulates in the nephritic region, particularly the kidney and brain, while Pb accumulates in the blood and bones, increasing their vulnerability much beyond the time of direct contact [9]. Fish intake, dental amalgam use, preservatives in immunizations, drug take-home vulnerability from occupational worker parents can all result in prenatal and postnatal Hg exposure. ASD has been connected to the neurotoxicity of Hg through several pathways, including reactive oxygen species (ROS) generation altered Ca^2+^ mitochondrial dysfunctions, disorganizing cytoskeleton, and glutamate excitability. Hg susceptibility in lower doses either pre and early postnatal produces reduced memory, attention, and language as well as deficient skill and lower interquartile according to some, though not all studies. People who have at least one Apolipoprotein E4 (*APOE4*) allele may exhibit significant social dysfunction and cognitive decline. Because APOE is a lipid transport protein involved in neural repair, the *e4* allele may inadvertently increase the risk of Hg-induced brain damage. This emphasizes the interplay between toxicants and genetically sensitive backgrounds rather than the effect of the e4 allele [23,27].

#### 2.3.2. Organophosphate

Pesticides known as organophosphates (OP) are widely employed in agricultural, as well as industrial, residential, and commercial settings. Since the ban on organochloride, organophosphate is the insecticide most used in agriculture. These chemicals can cause vulnerability both during pregnancy through the placenta and after birth through the intake of breast milk and food. Prenatal exposure to OP has been related, on a behavioral level, to abnormal conduct in newborns, slowed neurodevelopment, poor attention span, and widespread growth issues. Instead, prenatal OP vulnerability results in poor cognitive potential, along with deficits in working memory and emotive reasoning, as observed in children between the ages of 6 and 9 years [27]. The neuroanatomical position of the animal brain may parallel this mediocre shortcoming. Children of rural women exposed to organochlorine insecticides during the period of prenatal CNS development have been found to have a higher risk of developing ASD [27].

### 2.4. Congenital Viral Infection

The infectious disease most associated with increasing the risk of autism after a congenital infection is considered to be rubella. Prenatally infected children exhibit these multisystem abnormalities caused by the viruses. Huge CNS manifestations can take many different forms, from the rare absence or malformation of myelin in the cortex to cortical malformation indicative of a migratory defect. According to the literature, 7.4% of people in the autism spectrum have a high risk of developing a spatial brain infection if it starts to develop around week eight after fertilization [10]. One abnormality, along with others like cardiopathy, defects in the ability to hear, and mental retardation, were quickly linked to symptoms of Autism. Signs of psychiatric medical symptoms in cognition caused by prenatal rubella infection were discovered to change considerably over time. Based on numerous case reports, there is confirmation of prenatal cytomegalovirus (CMV) infection in increased cases of autism [11]. It is unknown how much of autism is caused by direct viral damage, how much is caused by the powerful immune response triggered by herpes viruses like CMV, or how much is caused by the type and location of brain malformation that develops particularly quickly in congenital CMV infection [27]. Inflammation’s function in the etiology of neurodegenerative diseases has been reorganized more frequently over the past ten years [28]. Even after trauma, neuronal and glial wounds continue to activate for a considerable amount of time. From this point of view, neuroinflammation is just a mark of something that happened before the neurons in the common region, marking the reality of the situation. However, a growing amount of evidence over the last 5–10 years has refuted this prior perspective and suggests that neuroinflammation plays a more active role in the beginning and development of disease [29].

### 2.5. Other Factors

Incidents that may occur in utero appear to be another factor contributing to the evolution of ASD. One other conclusion from the twin study highlights the importance of the prenatal environment in the development of the fetus [30]. The autism compliance rate is significantly higher in twins than in singletons when the two develop in the same chorion sac during the pregnancy. There is a much higher prevalence or the possibility of spreading ASD among those who are genetically predisposed to them during the winter months of pregnancy, maternal infection in the second trimester, weight loss, and complications during delivery. The spread of autism or other mental illnesses among people at genetic risk is influenced by biological factors in one or more cases. The evolved brain during fetal evolution may be more susceptible to viruses due to genetic makeup.

One or more of these extremely unfavorable episodes may be related to obstetric delivery problems, such as a temporary oxygen shortage. These assaults may, in turn, demonstrate excessively abnormal brain structure, shape, or tissue, as well as improve brain chemistry and shape in humans. The alterations of ASD can generally be compared to four main groups of complications such as complications of pregnancy: bleeding, diabetes, and rhesus factor; atypical fetal growth and maturing, issue of delivery asphyxia, and maternal infection, etc. [31].

## 3. Current Therapies for ASD

The current situation shows that autism cannot be treated, although numerous interventions involving adult children have been demonstrated to be effective. It reduces the intervention’s side effects, improved the child’s functioning capacity, and continued to develop their living skills. Due to the unique ways that autism impacts everyone, patients with ASD tend to face novel difficulties and have weaker social skills, cognitive abilities, and behavioral traits. As a result, children with autism should continue to have their health evaluated as part of their treatment plan. It is frequently difficult to determine whether a child’s conduct is connected to autism, which is brought on by a different set of health issues. Head punching may be a symptom of autism or a sign that a youngster has earaches or headaches; a physical examination is necessary to determine the cause. Monitoring a child’s health development entails paying attention to both the child’s physical and mental health in addition to symptoms associated with autism [32].

### 3.1. Medications

There are no pharmacological treatments that can alter the signs and symptoms of autism, although different medications can help to control symptoms that are similar to autism. For instance, a doctor may prescribe an antipsychotic, an antidepressant, and an anxiety medication if your child is hyperactive or to treat other behavioral problems. Be-fore prescribing the patients any new medications, it is recommended to first prescribe them dietary supplements for health care. There may be interactions between supplements and medications that have harmful side effects. Some vitamins and drugs may interact, leading to potentially harmful adverse effects [33]. Ginger, cranberry, valerian, raspberry leaf, chamomile, peppermint, rosehip, thyme, fenugreek, green tea, sage, and aniseed are among the herbal remedies most frequently used during pregnancy. Other herbal remedies used during pregnancy include eucalyptus, tenaadam (*Ruta chalepensis*), damakess (*Ocimum lamiifolium*), feto, and others. Not only tis but some other herbs that are used during pregnancy include dogonyaro (*Azadirachta indica*), palm kernel oil, bitter kola, and garlic. Higher doses of these herbs may lead to the untoward effects during the pregnancy or may disturb the fetal development [34]. The risk of both early and late spontaneous preterm delivery may be significantly decreased by consuming garlic during pregnancy. Numerous studies have also described the advantages of peppermint oil inhalation for treating nausea and vomiting [12]. Women who take excessive amounts of calcium and iron-rich supplements during pregnancy may not absorb zinc as well. ASD can be effectively treated with folic acid intake, but issues with folate metabolism and folic acid overload during pregnancy have been linked to the emergence of ASD symptoms in offspring [13].

### 3.2. Behavior and Communication Therapy

Numerous studies address the severity of the social, linguistic, and behavioral difficulties linked to autism spectrum illnesses [14]. A few studies focus on reducing problematic behavior, instruction, and skill novelty. Additional events emphasize teaching patients how to behave in social situations or how to communicate better with others. By using a reward-based system for motivation, application behavior analysis can assist children in learning novel skills and generalizing them to different contexts [29]. Children with ASD often respond competently to huge layout events of education. Such events successfully usually incorporate a team of specialists and a diverse response to communication, social skill, and behavior. Children who have received exhaustive separately to individuals’ behavioral involvement frequently appear to have better advancement. Depending on the needs of the autistic child, therapy will improve speech to change communication skills. Practical therapy that adapts to daily physical therapy lessons to change action and equilibrium may be beneficial. A psychologist may be troubled by issues with conduct and direction [35].

## 4. Inflammatory Pathways and Immunoinflammatory Link of ASD

**Figure 2:**
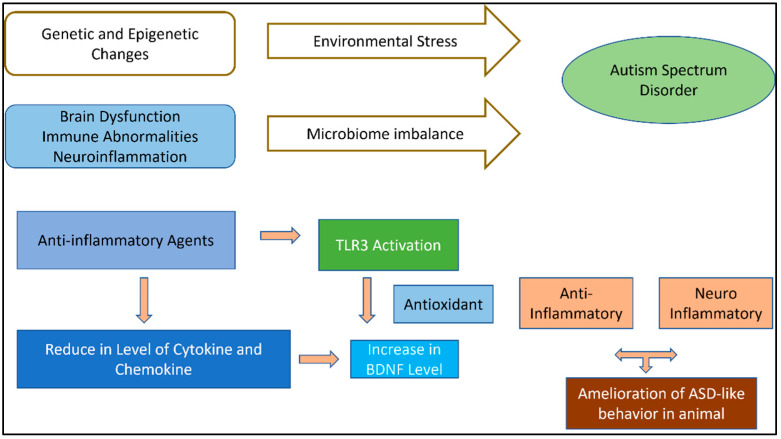
Pathogenetic mechanisms of ASD and related anti-inflammatory agent action with potential therapeutic effects illustrated in ASD animal models.

A comprehensive theory on all conceivable immunological pathways that have a significant impact on the epidemiological theory of ASD is outside the purview of this review. Prior to focusing on a feasible anti-inflammatory therapy for the treatment of ASD, we would like to draw attention to the biomarkers that are most crucial to an anti-inflammatory therapeutic strategy. A component of the inflammatory pathway was suggested by several rules of confirmation to be involved in the basic mechanisms of ASD development. Offspring of adult animals with infectious ASD symptoms during pregnancy mimic changes in behavior and human physiology in the rodent model of ASD. Pro-inflammatory biomarkers tend to increase while anti-inflammatory biomarkers tend to decrease, and some autistic people are encouraged to have improved inflammatory biomarkers (Figure 2). With regard to the available data, a sizable body of literature has established the character effects of inflammatory treatment of ASD that are associated with challenge therapy of adjuvant as separately [36].

Pathogens, damaged cells, poisonous substances, and other causes can all cause inflammation, which is an immune system’s biological reaction. Inflammatory signaling pathways, most frequently the Nuclear factor kappa B (NF-κB), mitogen-activated protein kinase (MAPK), and Janus kinase- signal transducer and activator of transcription (JAK-STAT) pathways are activated by both viral and non-infectious agents, cell injury, and inflammation [37].

### 4.1. NF-κB Pathway

The NF-κB family controls the regulation of several genes involved in inflammation, immunological responses, anti-apoptosis, and cell proliferation. More than 500 genes related to inflammation have been shown to be activated by the NF-κB family, which in turn triggers the release of cytokines necessary for inflammation. Some of these cytokines, such as IL-1β and TNF-α, cause the NF-κB to become active, creating a positive feedback loop that, if NF-κB becomes abnormally or continuously active, has the potential to cause chronic and excessive inflammation. The neurological system seems to be where the ubiquitous functions of NF-κB in neuroprotection, apoptosis, and inflammation are most pronounced. The consequences of NF-κB activation on gene expression vary depending on the stimulus that activates it, the type of cell that it targets, and the microenvironment. According to previous research, NF-κB activation in glial cells may result in the loss of neurons, but activation in neurons may increase the likelihood of their survival. Increased expression and enriched NF-κB signaling has been found in some studies performed on the peripheral blood samples and postmortem brains of ASD patients, as well as in studies on animal models [38].

IL-17A cytokine produced by a variety of immune cells, engages in both acute and chronic inflammatory reactions by upregulating the expression of pro-inflammatory genes via the NF-κB and MAPK signaling pathways. MAPK containing three subsequently activated protein kinases that are key components of a series of vital signal transduction pathways that regulate processes such as cell proliferation, differentiation and cell death in eukaryotes [39]. Evidence already in existence suggests that altering the downward signals coming from IL-17RA in response to IL17A may play a significant role in the neuroinflammation seen in ASD patients. When investigated the IL-17A/IL-17RA associated signaling in monocytes of ASD patients, they discovered elevated IL-17RA expression together with NF-κB and inducible nitric oxide synthase (iNOS) in these patients’ monocytes [40].

### 4.2. JAK/STAT Pathway

It has been shown that the JAK2/STAT3 signaling pathway activates and releases inflammatory cytokines such IL-1, TNF-α, and IL-6 [41]. Cognitive impairment could result from abnormal synapse construction and function brought on by a shift in the c-Jun N-terminal kinase (JNK) pathway in response to IL-1. JAK/STAT signaling cascades are triggered by IL-4. Interferon-gamma (IFN-γ) concentration was markedly elevated in the brains of those with ASD. For T-cell activation and differentiation in autoimmune CNS inflammation, IL-9 is crucial. Children with ASD had higher levels of IL-9, which shows that IL-9 may have a role in the emergence of ASD. Children with autism may exhibit behavioral changes as a result of peripheral immunological activation [42].

### 4.3. MAPK Pathway

IL-17 also activates the MAPK pathways, which include the extracellular signal-regulated kinase (ERK), p38, and JNK pathways. The ERK/MAPK signaling is known to play a critical role in brain development, as well as in learning, memory, and cognition [43].

### 4.4. TGF-β Signalling Pathway

Increased TGF-β levels through its receptors TGF-β R1/R2 and putative neuropilin 2 receptors may cause altered brain-derived neurotrophic factor (BDNF)/Tropomyosin receptor kinase B (TrkB) processing such that neurons produce increased pro-BDNF and less full-length TrkB. BDNF is a key molecule involved in plastic changes related to learning and memory processes, while TrkB is well known for its function during the development of nervous system [44]. Decreased TrkB signaling combines with decreased fibroblast growth factor (FGF)8/17 signaling to produce additive decreases in PI3K/Akt/mTOR (mammalian target of rapamycin) signaling. TGF-β is a transcriptional activator of BDNF and TrkB [45], but IL-1β inhibits BDNF signaling and decreases BDNF levels. Therefore, aberrant neural stem cell growth and differentiation may be caused by inflammation-regulated BDNF signaling through changing the Wnt/-catenin signalling pathway [46].

In addition to elevations of specific cytokines, their combinations may be related to specific symptoms and their severity in ASD. In previous studies elevations in IL-1β, IL-6, and reductions in TGF-β were found children with ASD who had more severe symptoms [6]. Supporting those results, we found that a combination of reduced IL-6 and IL-1α and elevated IL-17 may help differentiate children with no ASD symptoms from those with ASD symptoms. Therefore, the aim of this study was to determine the expression levels of IL-1β, IL-1α, IL-4, IL-6, IL-17, TNF-α, and TGF-β in peripheral blood mononuclear cells of children with ASD and healthy controls and determine the correlations between cytokine levels and clinical symptoms to determine their contributions in ASD [47].

## 5. Significance of Natural Anti-Inflammatory Agents in ASD

**Figure 3:**
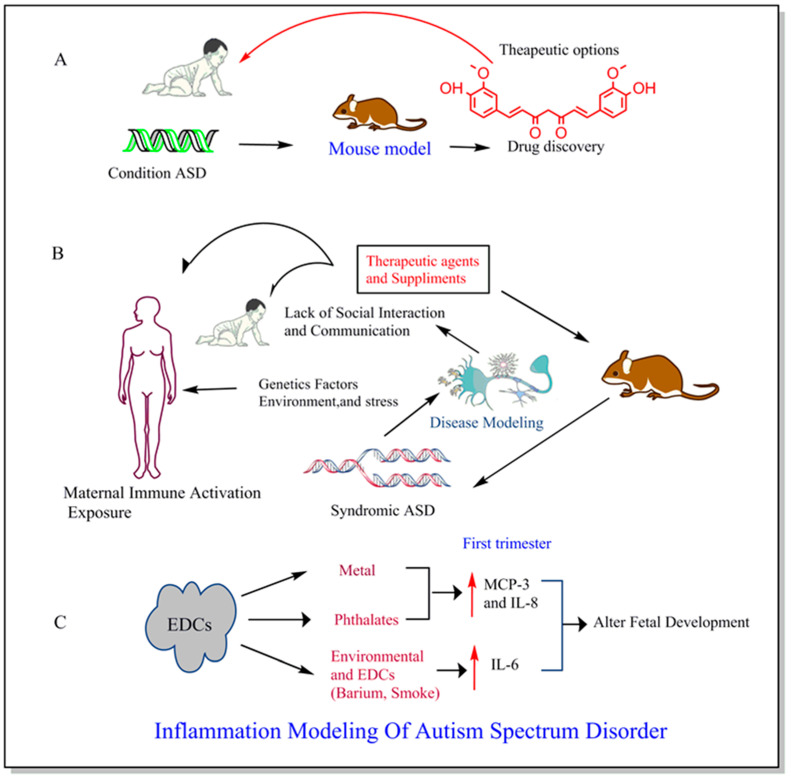
Historically, ASD Symptoms has been studied using mouse models leading to the drug discovery from a particular therapeutic link (**A**). Factors like genetic, environment, and stress causing physiological changes and the complex formation of disease in both human and mouse model and anti-inflammatory agents and supplements can be given as the potential treatment for ASD during early pregnancy (**B**). The maternal exposure to common endocrine-disrupting chemicals (EDCs) such as metal, phthalate, barium, and smoke contributes to the changes in immunoinflammatory markers mostly during first trimester resulting into alterations in fetal development (**C**) [52].

The importance of natural anti-inflammatory drugs in ASD has recently come to light. These drugs may have positive effects on autistic patients and greatly reduce maternal infection and brain inflammation in the etiology of neuropsychiatric disorders. There are a variety of naturally occurring anti-inflammatory agents’ studies so far that showed promising results against ASDs through their antioxidant and anti-inflammatory actions. The inflammatory response is the coordinated activation of many signaling pathways that keep track of the amounts of inflammatory mediators in native tissue cells and inflammatory cells from the blood. Many chronic diseases, such as cancer, diabetes, rheumatoid arthritis, cardiovascular disease, and bowel illness, have straightforward pathophysiology that involves inflammation. Although inflammatory response strategies vary depending on the place of the body, they are all based on common mechanisms that can be pictured as follows: Inflammatory pathways are engaged, inflammatory markers are generated, inflammatory cells are attracted, and cell surface pattern receptors detect harmful stimuli [48]. Studies have demonstrated the beneficial effects of natural anti-inflammatory agents in an animal model of ASD and autistic children by modulating the signaling molecules of inflammatory pathways (Figure 3). Sachdeva al. demonstrated that curcumin showed beneficial effects in valproic acid-induced-autism in rats by decreasing the IL-6 level [49]. Luteolin, an anti-inflammatory agent, also improved the behavioral symptoms in autistic children by reducing IL-6 and TNF-α level [50,51]. However, more research needs to be done to further investigate the potential beneficial effects of the naturally occurring anti-inflammatory agents for the prevention and treatment of ASD.

## 6. Preclinical Studies Targeting Inflammatory Pathways

Animal models of the circumstances of biology-based pharmaceutical treatment of ASD clearly show a critical function for ASD (Table 1). However, ASD is a unique human illness, and there is not a single rodent model that can replicate every essential ASD trait. It can be used to create animal models that provide insights into the brain mechanisms that control behaviors related to ASD and the inheritance of genetic traits that are comparable to ASD [53].

Dr. Leo Kanner originally discussed the idea of a link between pro-inflammatory cytokines and ASD in macrophages in 1943. The notion was founded on the fact that macrophage-produced cytokines affect ASD symptoms and the consequences of social and emotional disorders on the brain when administered to active individuals. The risk of ASD is significantly increased by prenatal exposure to the rubella virus, herpes simplex virus, cytomegalovirus, or viral meningitis [56]. The non-genetic etiology of autism is infection with germs and viruses prior to delivery. An analysis of all Danish infants born between 1980 and 2005 revealed a higher risk of ASD in cases where mothers were admitted to the hospital for viral illnesses at the beginning of pregnancy or for bacterial and viral infections at the end of the first trimester [57]. Children’s chances of developing ASD are increased when their mothers have celiac disease or rheumatoid arthritis [58]. Clinical and epidemiological research has correlated early pregnancy maternal infection with the fetus to autism. Recent research suggests that prenatal infection viral-like immune responses can cause permanent hyper- and hypomethylation at signaling genomic regions, which further disrupts the transcription of downstream target genes and may play a role in ASD [59].

The level of inflammatory cytokines and the dysregulation of mothers are likely factors contributing to the offspring’s delayed neurodevelopment, according to many findings from studies conducted on both humans and animals [60]. Crossing typically worsens behavioral phenotype and increases the probability of developing ASD [61]. Aripiprazole and risperidone are the most common psychiatric medications given to children with ASD to reduce their disruptive and violent behavior, although these medications have little effect on the fundamental symptoms of ASD. Studies have called into question the beneficial effects of psychotropic substances and have highlighted fast adverse reactions such as weight gain, sedation, tremor, signal abnormalities, and drooling. Growing polypharmacy has led to an unacceptable danger of drug interactions [62].

**Figure 4:**
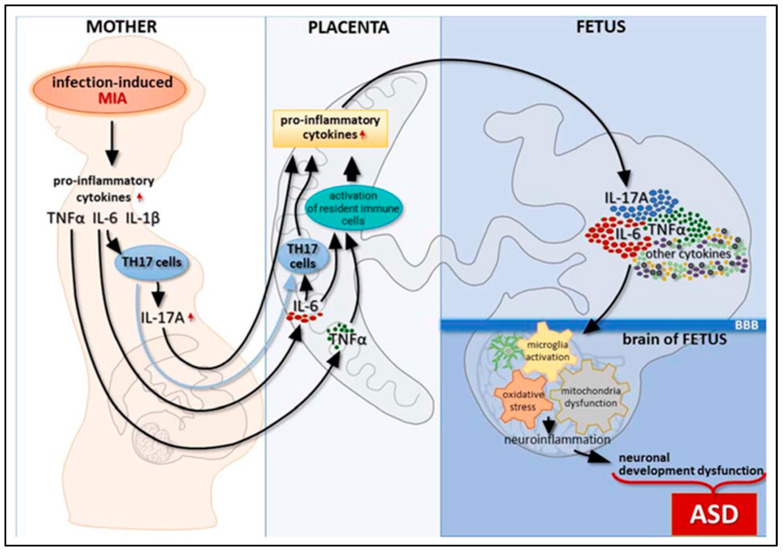
The relation between inflammatory pathways and immunoinflammatory link of ASD during pregnancy. Adapted from Zawadzka, A.; Cieślik, M.; Adamczyk, A. The Role of Maternal Immune Activation in the Pathogenesis of Autism: A Review of the Evidence, Proposed Mechanisms and Implications for Treatment. *Int. J. Mol. Sci.* **2021**, *22*, 11516 [38].

In addition to reduced social contact and repetitive activity, altered ultrasonic vocalization, a deficit in learning and memory, and pre-impulse inhibition, they indicate behavioral abnormalities related to neuropsychiatric diseases [63]. The use of behavioral analysis for various CNS variants is becoming clearer. A model has enabled a convention joint to assess behavior, reinforcement by individual documentation, and utility replacement in the anatomical regions located in CNS under the influence of humans. The injection of a maternal action is essential to the mechanisms of MIA [64]. TNF-α, IL-17a, IL-6, and IL-1 are a few examples of pro-inflammatory cytokines (Figure 4). Anti-inflammatory cytokine IL-10 overexpression is sufficient to protect the behavioral phenotype and is associated with neuropsychiatric diseases [65]. However, in the absence of IL-10, MIA [66] promotes aberrant behavior in the adults. Thus, the equalization of cytokines may be just as significant for the development of the fetal brain as any specific chemical. The maternal fluids, placenta, amniotic fluids, and fetal brain all have higher cytokine concentrations after immune system activation [67]. In both rodents and non-rodent primates, the changes in cytokines are region-specific and accompanied by neuropathological abnormalities, with the long events’ effects into adulthood.

The longitudinal effects are suggested to be associated with precise alterations in cytokine receptor distribution, or those that act as the other immune mediating process. Microglia, which act as resident CNS macrophages, are another example. Microglia plays a significant processing and neuromodulator role in neurogenesis, apoptosis, neuronal migration, and synapse remodeling [68]. MIA exposure to prenatal microglial expression causes other cytokines to be released and the downstream effects of the magnification factor as well as antioxidants to be activated. As a result, the actions on microglial cells have a long-term effect and may help to fuel the development of an inflammatory intermediate with neurotoxic effects in adults [69].

According to Bertolus et al. (2018), MIA leads to an increase in pro-inflammatory conditions in the developing fetal brain, which has wide-ranging effects on gene expression and genetics of neurons as well as excitotoxicity-induced damage to developing neurons. While excessive inflammation appears to increase the likelihood of abnormal cortical evolution, cytokines involved in the pathogenesis of MIA and autism, including IL-6, IL-TNF-, IL-1, and IL-17, are less attentive to TNF-α and IL-6 and play a physiological role in the proliferation of neuronal cells, differentiation, and abidance [70,71]. One of the important cytokines exposed in MIA is IL-6 in conjunction with IL-17′s downstream signaling. Pro-inflammatory cells’ stability is altered by cytokines in IL-6, which also controls placental T-cell behavior toward them. To clock the mother’s abandonment of the fetus, the placenta is inside an upsurge in regulatory T-cells above the pro-inflammatory Th17 cells at baseline. When MIA occurred, the balance shifted in favor of increased pro-inflammatory Th17 activity and decreased production of regulatory T cells [72]. When T cells are activated by IL-6, they produce IL-17, which is only found in the placenta and decidua [73].

When given to pregnant mice in the second trimester of pregnancy, poly (I: C) injections cause the serum level of IL-17a to increase. The offspring of the mouse subjected to increased levels of IL-17a had autistic features and displayed abnormal cortical patches. This was done to better understand IL-17a receptor upregulation in the fetal mouse brain. Numerous investigations have found raised IL-17a, which is consistent with human studies showing elevated IL-17a blood levels in ASD children and higher amounts in instances with more severe symptoms [74]. TNF-α is another cytokine involved in the pathophysiology of ASD. TNF-α participates in synaptic plasticity, memory and learning, and astrocyte-induced synaptic strengthening by activation of glutamate release at physiological levels [75]. Increased TNF-α release can intensify cytotoxicity caused by glutamate by preventing the absorption of glutamate into cells [76].

## 7. Clinical Studies Aimed at Targeting Inflammatory Pathways

The maternal offspring’s vagal system and control of their CNS are affected by maternal infection and activation pregnancies during studies. The MIA also intensifies the virus and bacteria in the mother’s gut, which collectively have the potential to affect the offspring’s microbiome (Table 2). The prenatal environment and delivery system occupy and influence the microbiota of the offspring [77]. MIA or maternal influence can cause behavior to worsen to a point where the fetus can move away from the placenta. The child’s hypothalamic-pituitary axis is overactive due to this program [78]. Although heredity has a significant role in the development of neuropsychiatric diseases, large-scale twin studies on ASD and Schizophrenia (SZ) reveal that environmental factors may also play a role [79]. Birth cohort studies have used archival samples or documented infection diagnoses made during pregnancy that is connected to specific information on the children in registry data since epidemiologic studies have difficulty characterizing individual exposures [40,80]. A “pathogen-free model,” or women with autoimmune illnesses, is another source of human data [81]. Independent risk factors for a variety of illnesses in offspring include ASD, SZ, attention deficit, and mood disorders, plus maternal Type 1 diabetes, rheumatoid arthritis, systemic lupus, and thyroid disease [82].

### 7.1. BDNF-TrkB Pathway

BDNF family of nerve growth factors, sometimes referred to as neurotrophins (NT), helps the brain develop both during pregnancy and after birth. BDNF plays a significant role in neuronal plasticity, neurotransmitter release, neuronal growth and survival, long-term potentiation and memory [84]. BDNF could be important biomarker to be investigated in the serum and blood of the autistic patients. Some studies have shown the increased level of BDNF in the serum and blood of neonatal autistic patients. However, few studies have shown the decreased level of BDNF in blood and brain tissues of autistic children compared to age-matched control or teenage children [85,86,87]. The downregulation of BDNF in AkT leads to a decrease in attention, which is a growth factor for Akt activities. We conclude that one of the underlying mechanisms that may be responsible for the pathophysiology of autism is the downregulation of the BDNF antiapoptotic signaling pathway in the brains of autistic individuals [88].

TrkB is most significant chemical in brain development, which includes Akt-mTOR, makes autistic individuals a candidate for engagement. A member of the tumor necrosis receptor superfamily called p75 neurotrophic receptor (p75NTR) shares structural similarities with the TrkB family of receptors and collaborates with other Trk family members to regulate the signaling of the TrkB receptor. By forming chimeric heteromeric complexes, different Trk family members and p75 NTR communicate with one another [89]. Due to its participation in numerous essential biological processes, has become a particularly attractive molecule of TrkB consequence surveillance in more recent times. TrkB functions as a receptor for NT-3 and NT-4 ligand and BDNF [44]. In addition to acting as a NT3 binding initiator, TrkC can also bind TrkB with lower affinity and regulate neurons. The homologous neurotrophin ligand activates the tyrosine kinase receptors, which thereafter dimerize with the unliganded monomeric form thought to be in balance with the phosphorylated dimeric state. While the exact cause of autism is unknown at the molecular level, studies in functional imaging, anatomy, and genetics show that synaptic modification and plasticity restrictions, as well as establishments that impede and improperly maintain the neural system, are what causes autism.

The clinical symptoms of ASD are thought to be fundamentally influenced by improved neuronal complex affinity (Table 2). Recent research on the etiology of ASD exposed compounds elaborates on synaptic plasticity and evolution. One of the BDNF receptors, TrkB, and proteins that are elaborate in their signaling pathways affect the stability and composition of the dendritic spine. The immune system, cellular homeostasis, and the transformation of the development component are all crucial to inflammation control. A group of children with autism was found to have decreased levels of TGF plasma and a relationship between the plasma level and behavior deterioration estimates [46].

### 7.2. JAK-STAT Pathway

Tyrosin kinase, a non-receptor family of the JAKs pathway, has four members: JAK1, JAK2, JAK3, and Tyk2. JAK1 is the first member of the family. The JAK family of proteins mediates the transmission of several cytokines and hormones, including immune system regulators, hormone growth factors, and many hematopoietic factors. The protein family’s structure is a JAK complex made up of seven homologous JAK and protein kinase domains. JAK3 is mainly found in hematopoietic cells and is one of three proteins that are hypothesized to be present throughout whole tissues. In addition to JAK pathway receptors, other phosphorylated signaling molecules with certain domains also bind cytokines and chemokines. JAK1 and STAT5 are raised in the blood peripheral mononuclear cells of autistic patients, which has major implications for the pathophysiology of diseases and opens the door to the potential use of certain chemicals as biomarkers for autism [90]. Signaling on the JAK/STAT pathway, which are the brain-derived neurotrophins that are involved in improving glial cell survival in the central nervous system, has been demonstrated to boost expression. We discovered that at that time, compared to controls, autistic children had a much higher level of JAK/STAT signaling. The limitations of IL-27 autoimmune encephalomyelitis are suppressed by central nervous system inflammation acting as a protective mechanism during inflammation.

### 7.3. mTOR Pathway

mTOR can control important cellular and molecular pathologies like protein production and mRNA translation. This regulation occurs in response to a wide range of external stimuli, including cytokines/chemokines, growth factors, hypoxia, and energy deprivation. Relevance of human illness in mTOR, which is increasingly recognized in oncology, neurology, immunology, and biotransformation/metabolisms, including autism. The role of the mTOR dysregulation signaling pathway in a subset specifically associated with autism and its contribution to our understanding of pharmacological and pathophysiological therapies for autism spectrum disorders are among the comprehensive molecular ASD on pathophysiology associated with the disorders that are accumulating evidence highlight the body [91].

### 7.4. NF-κB Pathway

The NF-κB route plays a major role in the processes of apoptosis, immunological activation, and inflammation. Nuclear kappa factor B can be activated based on the ability to express pro-inflammatory cytokines, which can include chemokines, adhesion molecules, and genes. The NF-κB is a complex player in the etiology of the targeting pathway of ASD, an indication of neuroinflammation that turns out to be crucial both as a marker for the illness treatment and as a selector for therapeutic interposition. A few theories have been developed on the role of NF-κB molecular signaling as the intersection of multiple elaborated pathways in autism. Inflammatory cytokines, both acute and chronic, are produced by the immune system and are involved in responses. Increased cytokine expression in conjugation with NF-κB/iNOS in patients with ASD has been discovered to be connected to autistic subjects’ monocytes. A rise in tyrosine evolution caused by up-regulation of iNOS through activation of the NF-κB signaling inflammatory targeting pathway may increase cytokine expression levels, which may contribute to neuroinflammation in ASD.

The biological and physiological immune system’s protective effects of nuclear factor kappa B activation are currently being outweighed by the detrimental effects of nuclear factor kappa B malfunction. Considerable progress has been made in comprehending the primary function and operation of NF-κB [88]. This study concentrated on the various experimental assay characterization of the antioxidant condition in autism by examining the redox transcription factors NF-κB in this serum at different levels, which increased in NF-κB serum fold 2.38 found in concentration on the ASD cases as differentiated to various mechanisms implication of the activation of NF-κB inflammatory signaling pathway in the etiology of ASD possibility due to the increasing burden of oxidative levels. A 2.2-fold increase in NF-κB DNA imperative project was found in ASD when compared with those from the age-matched group after the peripheral blood samples from 67 ASD subjects and 29 matched individuals were electrophoretically portable carry evaluated. This increase was also seen when the metaphysical experimental plan in innate immunity on the role and ROS condition of pathological in autistic patients was combined. Overall, it can be said that the connection between microglia substance and expression in different brain locations elucidates the prospective role of NF-κB in distinguishing the inflammation region of the brain on ASD [40].

### 7.5. Toll-Like Receptor Pathway

A fundamental function of the innate immune system is played by the family of proteins known as toll-like receptors (TLRs) [92]. LPS (lipopolysaccharides) is the component of bacterial and virus cell walls. Complex proteins are essential to the process of LPS recognition by TLRs [93]. The specialized proteins that bind LPS as a polymeric category medication during viral and bacterial infections create inflammation, which results in brain disruption. The conformational start of dimerization of the toll-like cytoplasmic receptor is altered by extracellular domains. Create a new scaffold with conformational modifications that will allow the post receptor’s adaptor protein to join a signaling complex [94]. The receptor, which produces a single domain trans-membrane receptor involved in pattern recognition, is often expressed in a variety of cells, including macrophages, dendritic cells, and numerous non-immune cells, including fibroblasts and epithelial cells [95]. There are many other types of pathways; here, we focus on the TLR route, which is responsible for both innate and adaptive signaling. Since only innate cells, such as monocytes, may express the TLR, it has been demonstrated that neuroinflammation involving this receptor takes priority. Immune system reactions to the exposure of autistics’ TLR4T monocytes and T-cells have been documented. TLR4 plays a significant role in the signaling of inflammatory mediators and oxidative stress in many immune cells. However, it is still possible to research the relevant toll-like receptor signaling in B cells in autistic people [96].

### 7.6. Mitogen-Activated Protein Kinase Pathway

Both threonine and serin are protein kinase types that are MAPK targets on direct cellular responses to several stimuli, including heat shock, mitogens, osmotic stress, and inflammatory biomarkers that regulate cell proliferation, differentiation, and death. An ERK1/2, p38 MAPK, and c-Jun N-terminal kinases are among the MAPKs that are connected with mammalian microtubules [78]. Every signaling process includes at least three MAPK components, which include MAPK kinase and MAPK kinase. Activate the MAPKKs and phosphorylate the MAPKKKs to activate the phosphorylated MAPKs. While inflammatory stimuli and stress activate JNK and P38, mitogen and other signals typically activate EKRs [97].

The ERK/MAPKs is another pathway that has been tied to autism which is involved in the various intracellular methods and techniques that are regulation of protein, which are the command in the growth of cells and trigger apoptosis. Genetic syndromes are including like neurofibromatosis type 1 and another name is Noonan syndrome. Autistic traits increased prevalence in autistic children RASopathies. There is the equality of PI3K/AKT and mTOR pathway, and various research of interest to treat targeting of ERK/MAPK for cancerous disease. The maturing of neuropsychiatric disorders has been studied in the targeting pathway of the ERK/MAPK in animal models. Micro deletion associated with mice is reconsidered to ASD in humans, which is used in the mice behavior deficits increase of ERK activity. Inhibitors of the ERK pathway are used to treat some animal behavior impairments in mice. The neurodevelopment in which the ERK pathway is affected occurs throughout a crucial time. Phosphorylation of ERK results in a momentary blocking of outcomes based on a postnatal day. Apoptosis in the forebrain caused memory loss and social problems in 6 mice [98].

## 8. Translational Studies Targeting Inflammatory Pathways (Patents)

Plant derived anti-inflammatory agents (Table 3) have contributed significantly in the development of drugs for various neuropsychiatric disorders including anxiety, depression [99].

Previous studies have also revealed the involvement of inflammation in the pathogenesis of ASD. Thus, targeting inflammatory pathways could be useful in developing new treatment therapeutics for the amelioration of autistic-like symptoms in children. Although few studies have done so far the investigation of natural anti-inflammatory agents for the treatment of ASD as reviewed recently by Pangrazzi et al. [100]. However, there are promising results that encourages the researchers to further explore the potential of these agents for the ASD prevention and treatment. In line with, only two translation work has been completed that demonstrated the effectiveness of natural anti-inflammatory agents against the inflammatory pathway signaling molecules. Most studied flavonoids including sulforaphane, resveratrol, quercetin improved the behavioral symptoms in the autistic patients, modulate pro-inflammatory or anti-inflammatory signaling molecules. These translational studies provided the basis to further explore the potential benefits of plant derived anti-inflammatory agents to treat either core or associated symptoms of ASD [101,102].

**Table 3:** Summary of translational studies of natural anti-inflammatory agents targeting inflammatory pathways.

S. No.	Patent No.	Natural Anti-Inflammatory Agent	Target	Method of Treatment	Reference
**1.**	US8937050B2	Glucosinolates Sulforaphane	Cellular stress response	Improvements in behavioral symptoms comprise one or more of the following: a decrease in Irritability, Hyperactivity, Stereotypy, and/or Inappropriate speech	[101]
**2.**	US20110104100A1	Reseveratrol Quercetin Glutathione	IL-10, IL-4, or TGF family members	Method of treating a pervasive developmental disorder comprising: Providing an agent with ability to inhibit host inflammatory reactions, Providing an agent or therapy capable of mobilizing endogenous stem cells, and Administering an effective amount of the agent with ability to inhibit host inflammatory reactions and an effective amount of the agent or therapy capable of mobilizing endogenous stem cells	[102]

## 9. Future Perspectives

Understanding the cellular and molecular signaling mechanisms underlying the anti-inflammatory pathway of autism spectrum diseases has advanced significantly. Targeting inflammatory pathways and inflammatory biomarkers in the treatment of ASD are covered in detail in this article. Genetic studies also support the idea that the immune system plays a part in neuropsychiatric abnormalities that result in numerous neurological diseases. The model must be explained by many theories, most of which center on how the environment and genetic variables contribute to inflammation. Natural anti-inflammatory drugs may be greatly beneficial in treating autistic patients and inflammation in the brain during pregnancy, which can affect the development of the offspring of adult animals. ASD is one of the many diseases in the inflammatory response. Our possible hypothesis is that environmental factors and genetic factors, which target the inflammatory pathway and response, are involved. Conventional rodents, which are immature at birth compared to human neonates, have been widely employed as animal models to examine the prenatal etiology of such outcomes in pregnant mice or animals at elevated risk of neuropsychiatric diseases.

## 10. Conclusions

Environmental and genetic factors can affect the inflammatory system, which can affect how the fetal brain develops during pregnancy. In conclusion, autistic mothers give birth to children who are underdeveloped and who can be treated with natural anti-inflammatory drugs. These substances have anti-inflammatory and neuroprotective effects on the developing brain. As a result, when these medications are provided to expectant mothers, the problem with the inflammatory pathways in both the mother’s and the fetus’s brain may be lessened.

## Figures and Tables

**Table 1:** Preclinical studies related to natural anti-inflammatory agents targeting inflammatory pathways for ASD treatment.

S. No.	Pathway	Anti-Inflammatory Agent	Target	Remark	References
**1.**	NF-κB	Resveratrol	IL-1β, TNF-α IL-17A	Blocks IL-1β & TNF-α resulted in a decrease in expression of NF-κB. Increases the levels of IL-17A.	[40]
		Palmitoylethanolamide & luteoiln	IL-1b, IL-6, TNF-α, NO, NF-κB	Significant decrease in NF-kB p65 levels in cerebral tissue from co-ultra PEA-LUT treated mice when compared to VPA mice.	[54]
**2.**	JAK/STAT	Luteolin Diosmine	IL-6, IL-12, IFN-γ	Reverses the behavior and neuropathological changes by inhibiting the STAT3 signaling pathway through IL-6. Inhibit IFN-γ-induced STAT1 activation & attenuate production of pro-inflammatory cytokines in cultured and primary microglial cells.	[41,55]
		Quercetine	IL-12	Inhibits IL-12 production and neural antigen-specific Th1 differentiation.	[55]
**3.**	ERK/MAPK	IL-17A antibody	IL-17A	IL-17 also activates the MAPK/ERK, p38, and JNK pathways. The ERK/MAPK signaling is known to play a critical role in brain development, as well as in learning, memory, and cognition.	[38,43]

ASD—Autism Spectrum Disorders; IL—Interleukins; IFNr—Interferons; TNF—Tumor Necrosis Factor; Poly (I:C)—Poly-Inosinic, Polycytidylic Acid; NF-κB—Nuclear Factor Kappa B; JAK/STATs—Janus Kinase/Signal Transducers and Activators of Transcriptions; MAPK—Mitogen-Activated Proteins Kinase.

**Table 2:** Clinical studies related to natural anti-inflammatory agents targeting inflammatory pathways for ASD treatment.

S. No.	Molecule	Targets	Patient Detail	Type of Study	Remarks	Reference
**1.**	Dietary Supplement: Luteolin, Quercetin & Rutin combined in a capsule (Neuroprotek)	IL-6 & 8 TNF-α	N = 50 (42M/8F) 4–10 years	Open-label trial	Inhibits histamine, IL-6, IL-8, TNF-α, & tryptase release from human mast cells.	[51]
**2.**	GSH, Vit. C, NAC	-	N = 24	Interventional	▲ GSH/GSSG ratio	[83]
**3.**	Luteolin formulation	IL-6 & 8 TNF-α	N = 40 (34M/6F) 4–10 years	Open-label trial	▼ Mean serum IL-6 and TNF levels	[50]
**4.**	Palmitoylethanolamide & luteolin	IL-6	A 10-year-old child	ATEC test	▼ IL-6, IL-1, & improves symptoms	[54]

ASD—Autism Spectrum Disorders; GSH—Glutathione; GSSG—Glutathione disulfide; NAC—N-acetylcysteine; ATEC—Autism Treatment Evaluation Checklist; ▼—decrease; ▲—increase; M—male; F—female.

## Data Availability

Not applicable.
